# Overcharging and Free Energy Barriers for Equally Charged Surfaces Immersed in Salt Solutions

**DOI:** 10.1021/acs.langmuir.1c02268

**Published:** 2021-12-01

**Authors:** Samuel Stenberg, Jan Forsman

**Affiliations:** Theoretical Chemistry, P.O.Box 124, S-221 00 Lund, Sweden

## Abstract

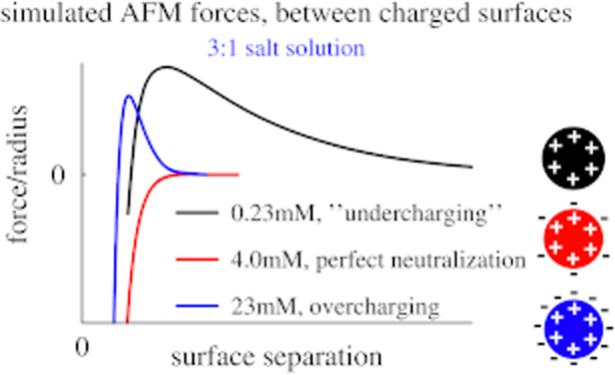

The stability of dispersions containing charged particles may obviously be regulated by salt. In some systems, the effective charge, as measured by the potential some small distance away from the particles, can have a sign opposite to the bare surface charge. If charge reversal takes place, there is typically a salt concentration regime within which colloidal stability increases with added salt. These experimental findings on dispersions have been corroborated by atomic force microscopy investigations, where an attraction is found at short separations. This attraction is stronger than expected from standard DLVO theory, and there has been considerable debate concerning its origin. In this work, we use simple coarse-grained models of these systems, where the bare surfaces carry a uniform charge density, and ion-specific adsorption is absent. Our hypothesis is that these experimental observations can be explained by such a simplistic pure Coulomb based model. Our approach entails grand canonical Metropolis Monte Carlo (MC) simulations as well as correlation-corrected Poisson-Boltzmann (cPB) calculations. In the former case, all ions have a common size, while the cPB utilizes a point-like model. We devote significant attention on apparent surface charge densities and interactions between large flat model surfaces immersed in either a 2:1 salt or a 3:1 salt. In contrast to most of the previous theoretical efforts in this area, we mainly focus on the weak long-ranged repulsion and its connection to an effective surface charge. We find a charge reversal and a concomitant development of a free energy barrier for both salts. The experimentally observed nonmonotonic dependence of colloidal stability on the salt concentration is reproduced using MC simulations as well as cPB calculations. A strong attraction is observed at short range for all investigated cases. We argue that in our model, all non-DLVO aspects can be traced to ion–ion correlations.

## Introduction

The intricate behavior of colloid and surface interactions in multivalent electrolyte solutions has been studied using both experimental^1−9^ and theoretical.^10−25^ approaches. These interactions are often of practical relevance, such as the condensation of DNA,^26^ but they are also of more fundamental interest. At weak electrostatic coupling strengths, that is, moderate surface charge densities, low ion valencies and high dielectric screening, such systems behave as one would expect from Poisson–Boltzmann and DLVO^27,28^ theory. At large separations, the electric double layers that develop near the particle surfaces generate repulsive forces. For low concentrations of monovalent salt, screening of the particle charge is inefficient, that is, the outer counterion layer is very diffusive (in an aqueous solution). This generates a long-ranged free energy barrier, which is often strong enough to prevent flocculation. As the salt concentration is increased, ionic screening becomes more efficient, thereby generating more short-ranged double layer interactions. This leads to weaker barriers, which destabilize the dispersion.

When ionic correlations become important, which in an aqueous solution usually require the presence of multivalent counterions, more interesting behaviors emerge. A well-known example is a short-ranged *correlation attraction* between like-charged colloidal particles, or surfaces.^11^ Another correlation-driven phenomenon is overcharging, or charge inversion, where the counterions overcompensate the bare surface charge. Charge inversion also means that the electrophoretic mobility is reversed. Overcharging has been found and studied in a range of different systems.^20−25,29−31^ In an experimental study by Van Der Heyden et al.,^32^ charge inversion was shown to occur in biologically relevant mixtures of salt. It should be noted that ion-specific adsorption can also generate charge reversal.^29−31,33^

Charge inversion also has a strong impact on colloidal stability. Sinha et al.^34^ and Ruiz-Cabello et al.^35^ used a combination of atomic force microscopy (AFM) and colloidal stability studies to demonstrate charge inversion in the presence of counterions with a valency exceeding two. Moreover, they showed that, in salt solutions containing ions with a high valency, colloidal stability displays a minimum at the isoelectric point, where the multivalent ions are able to perfectly neutralize the charged particles. At higher concentrations, charge inversion takes place, which has a stabilizing effect. These observations were corroborated by AFM measurements, with a repulsion at low and high concentrations of a multivalent salt, but a monotonic attraction at intermediate concentrations. This overall response was also found when the counterions are oligomeric^9^ or polymeric.^5^ We note that in the stability studies, the systems are canonical, that is, there is no infinitely large “bulk” solution, from (or to) which additional charge can be added. The AFM (or the surface force apparatus, SFA) setup, on the other hand, is grand canonical, in the sense that two macroscopically large surfaces are immersed in a solution large enough to ensure that the overall system contains more than enough counterion charge to neutralize the surfaces, even at minute salt concentrations. Nevertheless, at low enough concentrations, entropy will prevent this to occur.

There have also been theoretical studies on the relation between overcharging and free energy barriers. Turesson et al.^36^ utilized a grand canonical approach to study the interaction between two charged flat surfaces, immersed in a polyelectrolyte solution, where the polymers (or oligomers) were counterions to the surfaces. They found that even for moderately charged surfaces, overcharging occurs, with a concomitant double-layer repulsion between (effective) charge-inverted surfaces. In a subsequent work,^37^ they established that similar barriers are found if the polyions are replaced by spherical macroions with the same valency. One possible way to regulate the degree of overcharging, and thus the free energy barrier, is to insert a neutral midsection of the polyions.^38^

Overcharging will not be observed in a pure mean-field treatment, such as the Poisson–Boltzmann (PB) theory, but emerges when spatial correlation between the counterions is taken into account.^20−25^ The magnitude of the overcharging depends on the surface charge density. Hence, for two colloidal particles with a different magnitude of their charges, but the same charge sign, there is a possibility that the more highly charged particle is overcharged, while the other is not. This in turn would result in a long-range attraction, despite the ions having the same charge. A similar argument applies to oppositely charged particles, or surfaces, which might facilitate a long-ranged repulsion.^3,39,40^

In this work, we use a modified PB theory as well as grand canonical Monte Carlo (MC) simulations to calculate the interaction between charged surfaces in the presence of a multivalent salt at various concentrations. Using correlation-corrected PB (cPB) theory,^41^ we approximate spatial correlations between the counterions via a simple modification to the interaction potential between the multivalent cations. A great advantage of the cPB is that it gives direct access to the interaction free energy between the surfaces. It is also very simple and amounts to a tiny modification of the corresponding PB version, when formulated as a *classical* density functional theory (DFT).^13,42^ In contrast to most of the previous works on ion correlations and surface forces, we do not focus on the attraction at short range, but instead on how these correlations influence the interactions at long range. We calculate apparent surface charge densities (see below) and provide an explicit link between overcharging and the interaction between charged surfaces, at long range. We also investigate the range of the correlation attraction and discuss possible connections to surface heterogeneity. Our results are compared with experimental AFM data, on similar systems.

It should be noted that ion-specific behaviours,^29−31^ as well as effects from the molecular nature of the solvent,^43^ are outside the scope of this work.

## Models and Methods

Our model surfaces consists of two planar and infinitely large walls. These are separated by a distance *h* and carry a smeared-out charge, represented by a surface charge density σ_s_. A model aqueous salt solution fills the gap between the surfaces. Water is represented as a dielectric continuum via a dielectric constant ϵ_r_ = 78.3. In the simulations, the ion charges are centered in softly repulsive spheres, as described below. In the cPB treatment, however, ions are represented as point charges. The ions are in equilibrium with a bulk solution of a specified concentration.

The Coulomb interaction (free) energy between ions α and λ, separated by a distance *r*, is
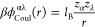
1where β is the inverse thermal energy, whereas the valency of ion α is denoted by *z*_α_. We define the Bjerrum length as *l*_B_ = β*e*^2^/(4πϵ_0_ϵ_r_), where *e* is the elementary charge and ϵ_0_ is the permittivity of vacuum.

In the *simulations*, all ions also repel each other by a short-ranged softly repulsive core potential, ϕ_s_
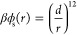
2where *d* = 4 Å. The reason to choose a soft, rather than hard, core is technical, as it will reduce noise in the pressure evaluations.

In the original PB theory, interactions between like charges are overestimated. This is because the mean-field treatment assumes a uniform charge distribution, and neglects the fact that ions of like charge will avoid close proximity. To correct for this overestimation, a modified interaction potential, ϕ_eff_^αα^, is used between like-charged ions, which effectively accounts for the “Coulomb hole” that like-charge ion correlations generate.^41^ Specifically, the repulsion between them is weaker than the Coulomb interaction, at separations below some value *R*_c_^α^
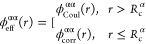
3where ϕ_corr_^αα^(*r*) is described as a tangent continuation of ϕ_Coul_^αα^(*r*) at *R*_c_^α^
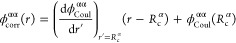
4

There are in principle other alternatives that also would improve the original formulation (PB).

*R*_c_^α^ should reflect the average nearest-neighbour distance between ions of like charge. If ion α is a counterion to the oppositely charged surfaces, then *R*_c_^α^ will be determined by “crowding” at the surfaces. In this work, we only include correlation-corrections between (usually multivalent) counterions, whereas correlations between the monovalent coions are neglected. In the original cPB formulation,^41^*R*_c_^α^ was defined as: , which is physically reasonable (see ref (41) for further motivation), but it is still an ad hoc quantification. Using this value, we arrive at cPB predictions that are in qualitative agreement with the simulation results. However, we noted that the simulated concentrations below which overcharging disappears are better reproduced (quantitatively) if *R*_c_^α^ is increased by 50%. Hence, in the main paper, we have used an adjusted value
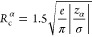
5

The results obtained with the original^41^ definition of *R*_c_^α^ are presented in the Supporting Information.

We shall by *x* denote the direction normal to the surfaces. The two flat walls are located at *x* = 0 and *x* = *h* and extend indefinitely in the (*y*, *z*) plane. They impose a hard-wall exclusion, ensuring that the *x* coordinate, *x*_*i*_, of all ions (center of mass) is located between these values, that is, 0 < *x*_*i*_ < *h*. In principle, there is also an external wall potential, *V*_ex_(*x*, *h*), originating from the smeared-out surface charge density, but cancellation effects remove the dependence on *x*. This is because an *y*, *z* integration, in combination with an electroneutrality constraint, gives us β*V*_ext_^α^(*x*,*h*) = −2π*l*_B_*z*_α_(σ_s_*x* + σ_s_(*h* – *x*)) = −2π*l*_B_*z*_α_σ_s_*h*, as the surfaces are equally charged. This appears to generate a separation-dependent potential, to be accounted for in grand canonical steps. However, because we always insert and remove electroneutral groups of ions, the surface charge potential needs not be considered in these processes and thus not in any MC steps (neither canonical nor grand canonical). In cPB, and PB, calculations, electroneutrality is maintained via a Donnan potential as has been previously described.^41^

Of central interest in this work is the interaction free energy per unit area, as the distance between the surfaces is varied. An advantage of the cPB (and PB) is that the free energy is directly available. In the simulations, periodic boundary conditions were applied along the directions (*y*, *z*) parallel to the surfaces. An external potential field, which was calculated from previously simulated ion density profiles, was used to account for the long-ranged interactions.^44,45^ In the grand canonical addition and deletion moves, an overall electroneutral group of ions was attempted to be added to, or removed from, the simulation cell. Thus, when performing an addition move of, for example, a 3:1 salt, one cation and three anions were attempted to be inserted at random positions. In the MC simulations, the free energies were calculated from the normal pressure. The pressure component normal to the surfaces, *P*_n_, was obtained by calculating the average *x*-projection of all interaction forces per unit area, acting across a chosen plane at *x*_p_, and then adding the corresponding ideal pressure, *P*_id_, where β*P*_id_ = ∑_α_*n*_α_(*x*_p_). The position of the plane, *x*_p_, can in principle be arbitrarily chosen, but the system symmetry does suggest either the location of a wall or the midplane between the surfaces as “natural” choices. The latter turns out to give superior statistical properties because the density usually is modest and slowly varying near the midplane. Hence, we have in almost all cases evaluated *P*_n_ at the midplane of the slit, but we do include a single comparison with the corresponding results at a surface. The normal pressure is then calculated for a range of surface separations. A cubic spline fit to these data results in an approximation of *P*_n_ as a function of separation, *P*_n_(*h*). By integrating this function, we arrive at the net interaction free energy per unit area, Δ*g*_s_
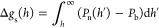
6where *P*_b_ is the bulk pressure, which in practice was estimated by the normal pressure at the largest investigated separation. Adopting the Derjaguin approximation,^46^ these interaction free energies are proportional to the force per radius measured in AFM, or SFA, experiments.

Our investigation will cover 2:1 and 3:1 salt solutions, at various concentrations. Some simulated and calculated data for a 1:1 salt solution are reported in the Supporting Information. Using the equilibrated ionic density profiles, we calculated an “apparent” surface charge density, σ_app_(*x*), defined as
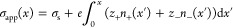
7where *n*_+_(*x*) and *z*_+_ denote the cation concentration and valency, respectively, with an analogous notation for the anions. The temperature was set to 298 K in all simulations and cPB (or PB) calculations.

## Results and Discussion

We shall focus our attention on two different systems: surfaces with σ_s_ = −0.01*e*/Å^2^, immersed in 2:1 salt solutions, and surfaces with −0.005*e*/Å^2^, immersed in 3:1 salt solutions. The results obtained for these systems are presented in separate sections below.

### 3:1 Salt, σ_s_ = −0.005*e*/Å^2^

**Figure 1:**
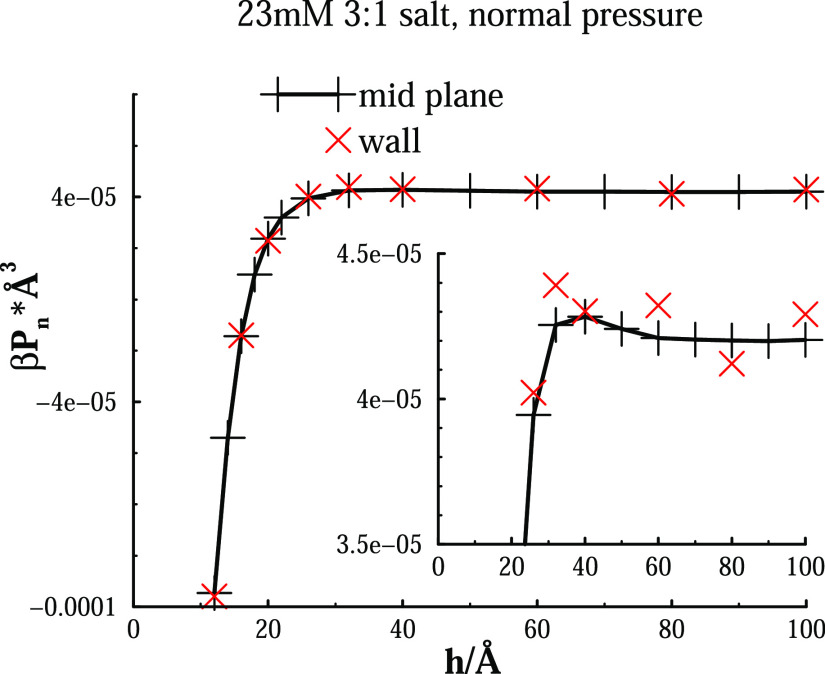
Simulated normal pressure curves. Model surfaces, with σ_s_, −0.005*e*/Å^2^ were immersed in a 23 mM 3:1 salt solution. Displayed are results from midplane (black plus signs) and wall (red crosses) evaluations. The line is a guide to the eye. Fewer data are reported for the normal pressure across a confining wall.

The cPB calculations produce interaction free energies directly as they are based on a free energy (classical) DFT. The simulation approach, however, generates normal pressures, *P*_n_, which need to be integrated in order to arrive at interaction free energies. In principle, one can calculate the normal pressure across any plane parallel with the confining surfaces, but two choices are arguably more natural than others: the midplane or one of the walls. The latter choice has two disadvantages compared to the former: the density at contact requires extrapolation, and the overall noise is quite substantial, as compared with midplane evaluations. Normal pressure data from both approaches are given Figure 1. The comparison is not completely fair because the wall pressures were calculated via density samplings from (roughly) the last 25% of the production simulations, but it is nevertheless clear that the midplane pressure calculations display considerably less noise.

**Figure 2:**
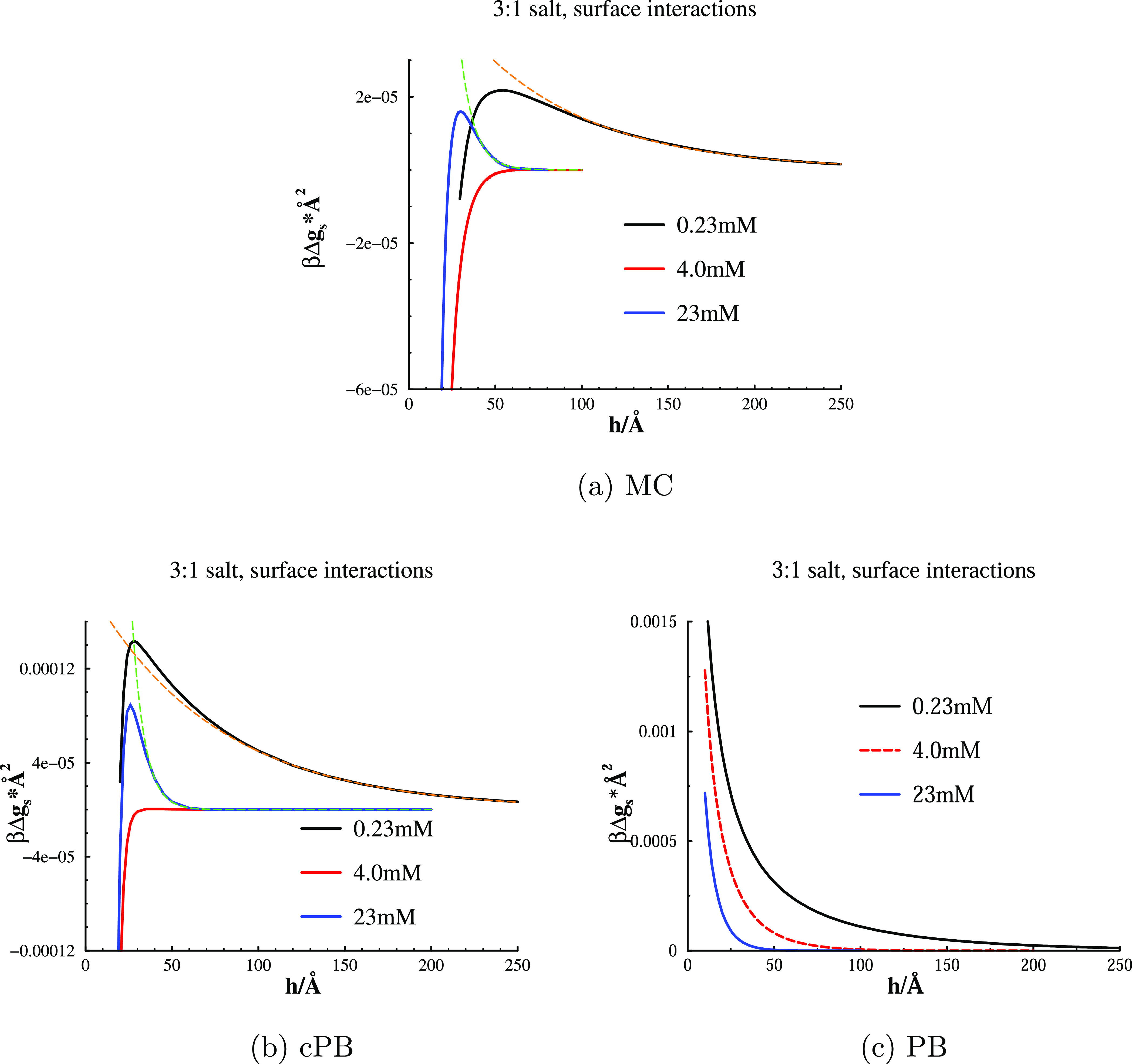
Interaction free energies per unit area with surface separation, as obtained from MC simulations [graph (a)], cPB calculations [graph (b)], and standard PB calculations [graph (c)]. Model surfaces, with σ_s_ = −0.005*e*/Å^2^, were immersed in a 3:1 salt solution. The dashed curves are numerical two-parameter fits, of the long-range regimes, to the expression βΔ*g*_s_ = *a*_0_*e*^–*a*_1_*h*^.

Simulated and calculated net interaction free energy curves, at various concentrations of 3:1 salt, are presented in Figure 2. In order to highlight the importance of correlations, we have also included a graph (Figure 2c) with data from standard PB calculations, that is, where the effective potential is equal to the Coulomb potential (*R*_c_ = 0). Simulations and cPB calculations display the same qualitative behaviour, with a substantial free energy barrier at low (0.23 mM) and high (23 mM) concentrations. At an intermediate concentration of about 4 mM, however, the barrier has vanished, resulting in monotonically attractive interactions (at least down to very short separations). The attraction results from well-established correlation effects, but curiously enough, so does the barrier that is gradually built up at concentrations above this threshold value. All of these effects are, as expected, lost with a pure PB treatment, as demonstrated in Figure 2c. In that case, the interaction is always monotonically repulsive, with a range that drops monotonically with salt concentration—a well-known DLVO result.

For a given surface charge density, there is obviously a threshold concentration of 3:1 salt (around 4 mM, when σ_s_ = −0.005*e*/Å^2^), above which overcharging occurs, and a free energy barrier is restored. It should be noted that this barrier has a shorter range than the barriers that are obtained at “undercharging” concentrations. This is a simple screening effect, resulting from an increased ionic strength. In fact, at very high salt concentrations, we expect the barrier to diminish as a result of ionic screening, although we have not investigated this regime.

The dashed curves in Figure 2 are numerical fits, of the long-range tails, to the expression βΔ*g*_s_ = *a*_0_*e*^–*a*_1_*h*^. We will discuss these fits in more detail below.

**Figure 3:**
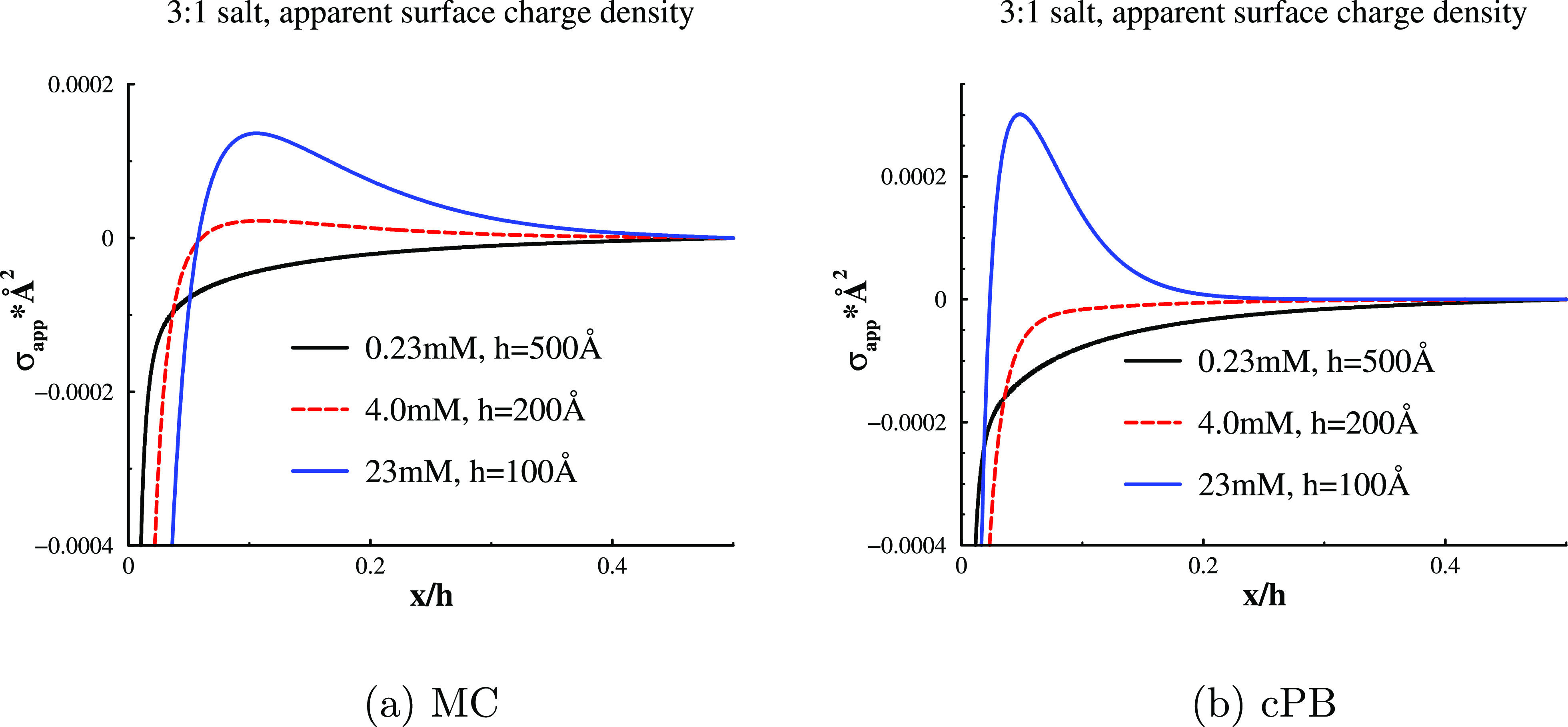
Apparent surface charge density plotted against a separation-normalized surface distance at various 3:1 salt concentrations. The separations were *h* = 500 Å (0.23 mM), 200 Å (4 mM), and 100 Å (23 mM), where the values were chosen large enough to achieve near bulk-like conditions at the midplane. The results from cPB calculations as well as MC simulations are shown. Conditions as in Figure 2.

We have already hinted that the free energy barriers are directly related to the surfaces being either under- or overcharged, that is, there is a remaining effective surface charge, when the charge contribution from counter- and coions in the vicinity surfaces have been accounted for. This is illustrated in Figure 3, where we plot how the apparent charge density varies with surface distance, at some large separation. We see how the bare surface charge is gradually being counteracted by a very diffuse layer at a low salt concentration, 0.23 mM, only achieving full neutralization at the midplane—which of course is demanded by electroneutrality. At a high concentration (23 mM), the bare negative surface charge is overcompensated by the trivalent counterions, resulting in an effective positive charge outside the primary (“Stern”) ion layer, that is, σ_app_ displays a local extremum near each surface. Further away from this extremum, the apparent surface charge density decays slowly toward a zero value at the midplane, in a manner similar to that at low concentrations. Finally, at an intermediate concentration, 4 mM, the σ_app_(*x*) profile is essentially flat, outside a primary counterion layer that just barely neutralizes the surface charge. In the two former cases, there is a long-ranged repulsion (see Figure 2), which is absent at 4 mM. Hence, we can relate a long-ranged slope of σ_app_(*x*) to a long-ranged double-layer repulsion. Moreover, the range of the slope itself is related to range of the repulsion, and at low ionic strengths, σ_app_(*x*) displays a very gradual drop, which is reflected in a correspondingly “stretched” free energy barrier. On the other hand, with a flat σ_app_(*x*) profile, resulting from perfect neutralization by the primary counterion layer, there is no such long-ranged repulsion and thus no free energy barrier.

The quantitative performance of cPB is far from perfect, but still satisfactory, given its simplicity. Predicted free energy barriers do vary somewhat too strongly with salt concentration. This is possibly related to the fact that the ions have no hard core (“size”) in the cPB treatment.

**Figure 4:**
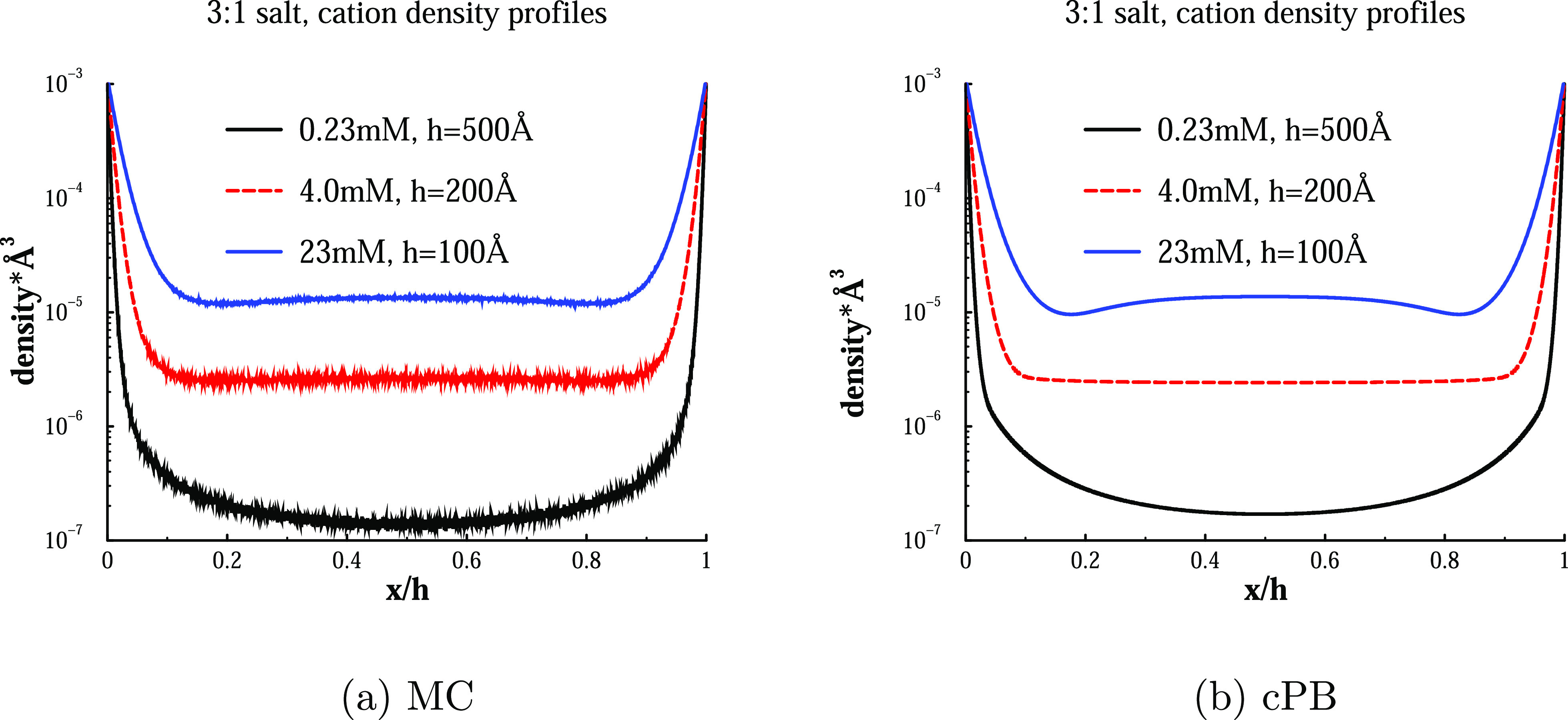
Cation density profiles at various concentrations of 3:1 salt. Conditions as in Figure 2.

These curves are in qualitative agreement with AFM measurements,^9,34,35^ although tetravalent counterions seemed to be required in order to establish a pronounced “overcharging barrier”. However, in Figure 4c in the work reported by Ruiz-Cabello and co-workers,^35^ there is an indication of a weak barrier building up at the highest investigated concentration, for trivalent counterions. We can also make crude quantitative comparisons between simulated and measured interactions. Applying the Derjaguin approximation, Δ*g*_s_ ≈ *F*/*R*, where *F* is the forces between curved surfaces of radius *R*, we can convert the maximum value, βΔ*g*_s_ ≈ 2.2*e*^–5^/Å^2^, at 23 mM (MC) to *F*/*R* ≈ 0.03 mN/m. Comparing with AFM data,^9,34,35^ we note that this is quite a reasonable value.

The occurrence of overcharging, or “charge reversal”, can actually be seen directly from the counterion density profile. Specifically, upon overcharging, this profile will display a local minimum near the surface. This is illustrated in Figure 4, where we again note a very nice agreement between the simple cPB theory and MC simulations.

At long range, we expect to recover the linearized PB predictions of an exponentially decreasing interaction free energy, decaying as *e*^–κ*h*^, where κ = [∑_*i*_(β*n*_*i*_*e*^2^*z*_*i*_^2^/(ϵ_r_ϵ_0_)]^1/2^. However, in the simulations, the formation of ion pairs, and higher-order clusters, may in principle lead to an increased effective Debye length, κ^–1^. We recall that the dashed lines in Figure 2 are numerical fits to the expression βΔ*g*_s_ = *a*_0_*e*^–*a*_1_*h*^. Such fits were performed at 0.23 mM (fitting range: 100–500 Å) and 23 mM (fitting range: 40–70 Å). These fits agree reasonably well with cPB as well as MC data, even though there is a small “intrinsic” error arising from the fact that we have set Δ*g*_s_ to zero at the largest separation, which of course is an impossible limit in the fitted expression, for any finite value of *a*_0_. The fitted decay lengths are 1/*a*_1_ ≈ 69 Å (MC) and 73 Å (cPB) at 0.23 mM and 1/*a*_1_ ≈ 8.1 Å (MC) and 7.2 Å (cPB) at 23 mM. These are in reasonable agreement with the expected Debye lengths, 1/κ ≈ 82 and 8.2 Å, respectively. The fact that the simulated estimate of the decay length actually is smaller than 1/κ suggests that ion clustering is rare under these conditions. We can proceed with attempts to connect the amplitude factor *a*_0_ to linearized PB approximations. In the latter case, a constant surface charge ansatz implies^47^ that βΔ*g*_s_ ≈ 2 * σ_eff_^2^*e*^–κ*h*^/(κϵ_r_ϵ_0_), where σ_eff_ is some “effective” surface charge density, which classically would agree with a value of σ_app_(*x*) at a position (*x*) just outside the “Stern layer”. If we relate the fitted *a*_0_ values to σ_eff_ in this manner, we end up with the following values at a low concentration (0.23 mM), where no overcharging occurs: σ_eff_ ≈ −0.000064*e*/Å^2^ (MC) and σ_eff_ ≈ −0.00011*e*/Å^2^ (cPB). Compared with the corresponding apparent surface charge density profiles in Figure 3, these values seem quite reasonable because they roughly coincide with a value of σ_app_(*x*) outside the steep regime near the surface (the “Stern layer”). However, applying the same procedure for our fits at a high concentration (23 mM), where there is a pronounced overcharging, generates quite high values: σ_eff_ ≈ 0.00095*e*/Å^2^ (MC) and σ_eff_ ≈ 0.0022*e*/Å^2^ (cPB). There may be several reasons for this, one of which probably is that the local maximum of σ_app_(*x*) varies with separation. There is of course also the aspect that the mean-field-based idea of a inner “Stern layer”, outside of which there is a “diffuse layer”, is not really compatible with correlation-induced overcharging.

**Figure 5:**
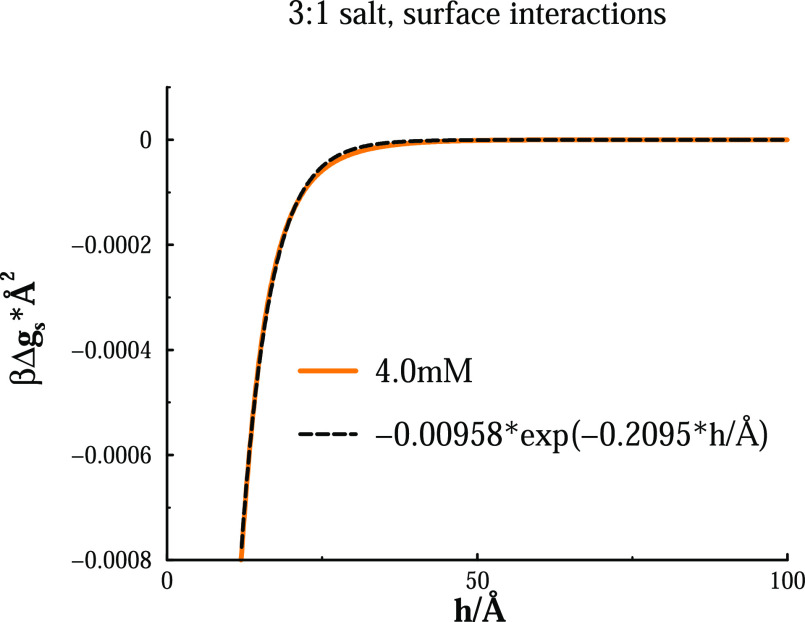
Fitting the simulated interaction curve for the 4 mM 3:1 salt (monotonically attractive) to an exponential expression, as indicated in the legend (dashed line).

Let us now turn our attention to the attractive part of the curves. Experimentally, it is common to fit this part to an exponential expression, the rationale being that it originates from attraction between charged patches on heterogeneous surfaces.^48−50^ In Figure 5, we have attempted such a fit for the 4 mM concentration, where the interaction is monotonically attractive. Now, if we apply the idea of a surface composed of correlating charged species, aligned on a lattice and separated a distance *a*, we expect an inverse decay length of [κ^2^ + (2π/*a*)^2^]^1/2^.^49^ Equating this to the fitted inverse decay length, 0.2095/Å, we arrive at *a* ≈ 21 Å. This is actually close to the average separation per trivalent ion, 24.5 Å, if we assume a perfect surface charge neutralization by these ions.

In principle, this lends support to the picture of an attraction resulting from correlations between two approaching surfaces carrying an adsorbed lattice-like monolayer of trivalent ions. However, we would argue that this amounts to an oversimplified view. In reality, the simulated distribution of trivalent ions on our model surfaces is far from lattice-like.

**Figure 6:**
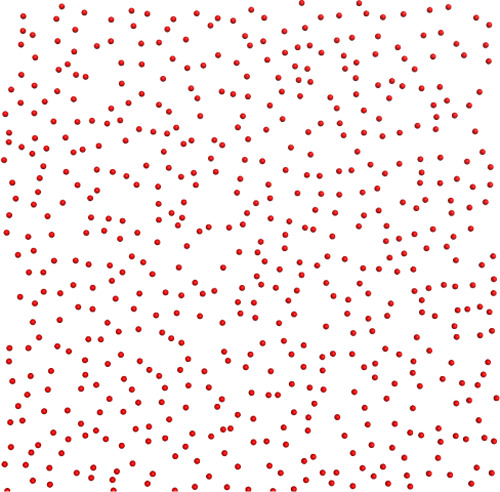
A simulation snapshot focusing on the distribution of trivalent ions (circles) very near one of the surfaces (4 mM 3:1 salt).

In Figure 6, we present a configurational simulation snapshot, where we have isolated one of the surfaces together with its primary layer of adsorbed trivalent ions (4 mM 3:1 salt). The distribution is obviously more “liquid like” than “lattice like”. Note, however, that this does not invalidate the notion of correlations between ions at opposing surfaces, but it does demonstrate that these surfaces (with adsorbed counterions) are not strongly heterogeneous, that is, there is no long-range order. We would argue that intersurface ion correlations form a subclass of the broader description “ion correlations”, that is, they are *not* competing theories, in our view. It should also be noted that the main ideas underlying the cPB formulation are based on correlations between adsorbed ions at a surface.

### 2:1 Salt, σ_s_ = −0.01*e*/Å^2^

We now proceed to corresponding investigations for a system with more highly charged surfaces, but with a reduced valency (+2) of the counterions. We expect qualitatively similar behaviours. It is nevertheless of interest to obtain quantified measures, which furthermore will allow us to test the cPB performance under quite different conditions.

**Figure 7:**
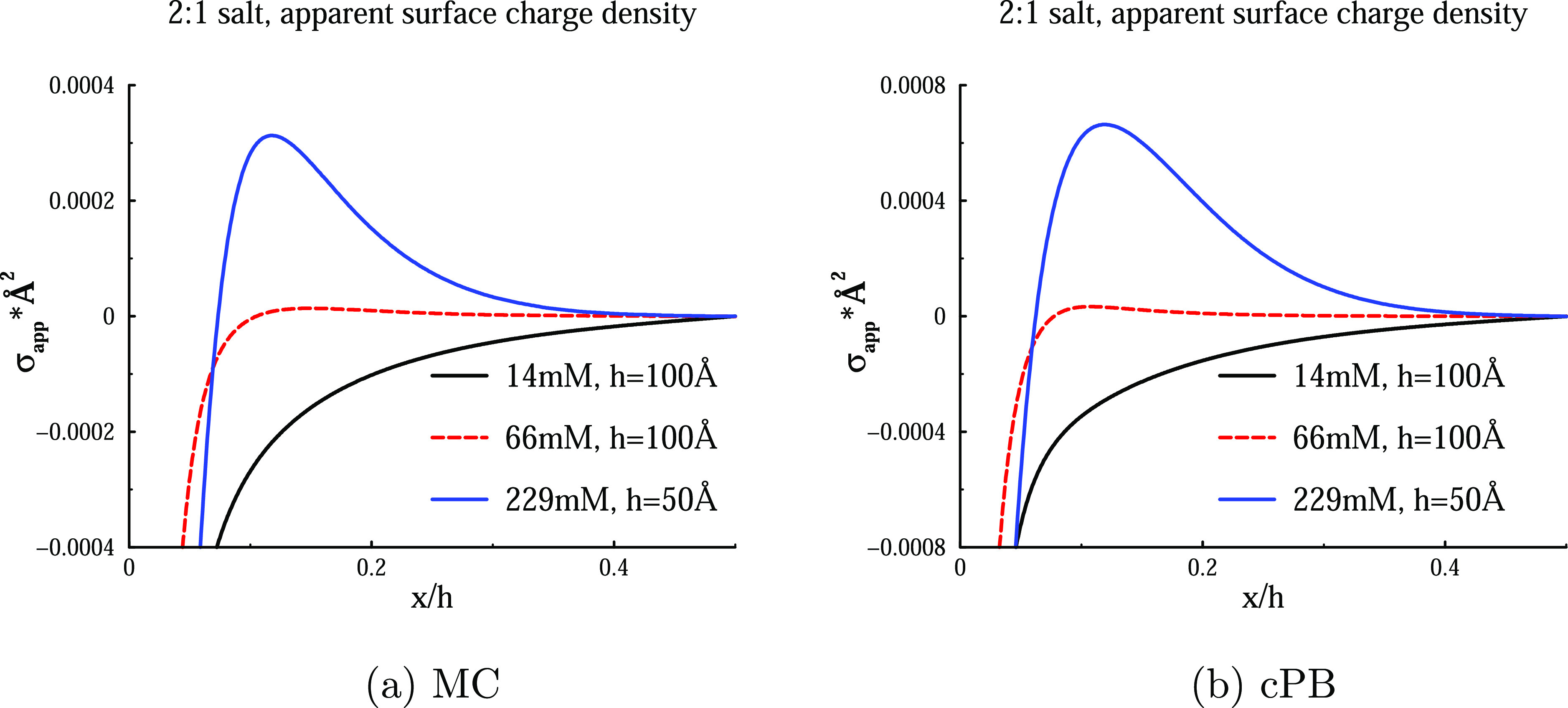
Apparent surface charge density, plotted against a separation-normalized surface distance, at various 3:1 salt concentrations. The separations were *h* = 100 Å (14, 66 mM) and 50 Å (229 mM), where the values were chosen large enough to achieve near bulk-like conditions at the midplane. The results from cPB calculations as well as MC simulations are shown. Model surfaces, with σ_s_ = −0.01*e*/Å^2^, were immersed in a 2:1 salt solution.

Profiles of the surface charge density are provided, for a range of different salt concentrations, in Figure 7. We note that even though the surfaces are more highly charged, a higher salt concentration is required to produce overcharging, as compared with the 3:1 salt solutions discussed above. The threshold concentration, at which the primary counterion layer is able to perfectly neutralize the surfaces, is in this case about 60–70 mM. An even higher surface charge density would see this value drop. As before, we observe overcharging at concentrations exceeding this value, and “undercharging” at lower concentrations. Weak ionic screening leads to a slow decay of σ_app_(*x*) toward the vanishing midplane value when the salt concentration is low.

**Figure 8:**
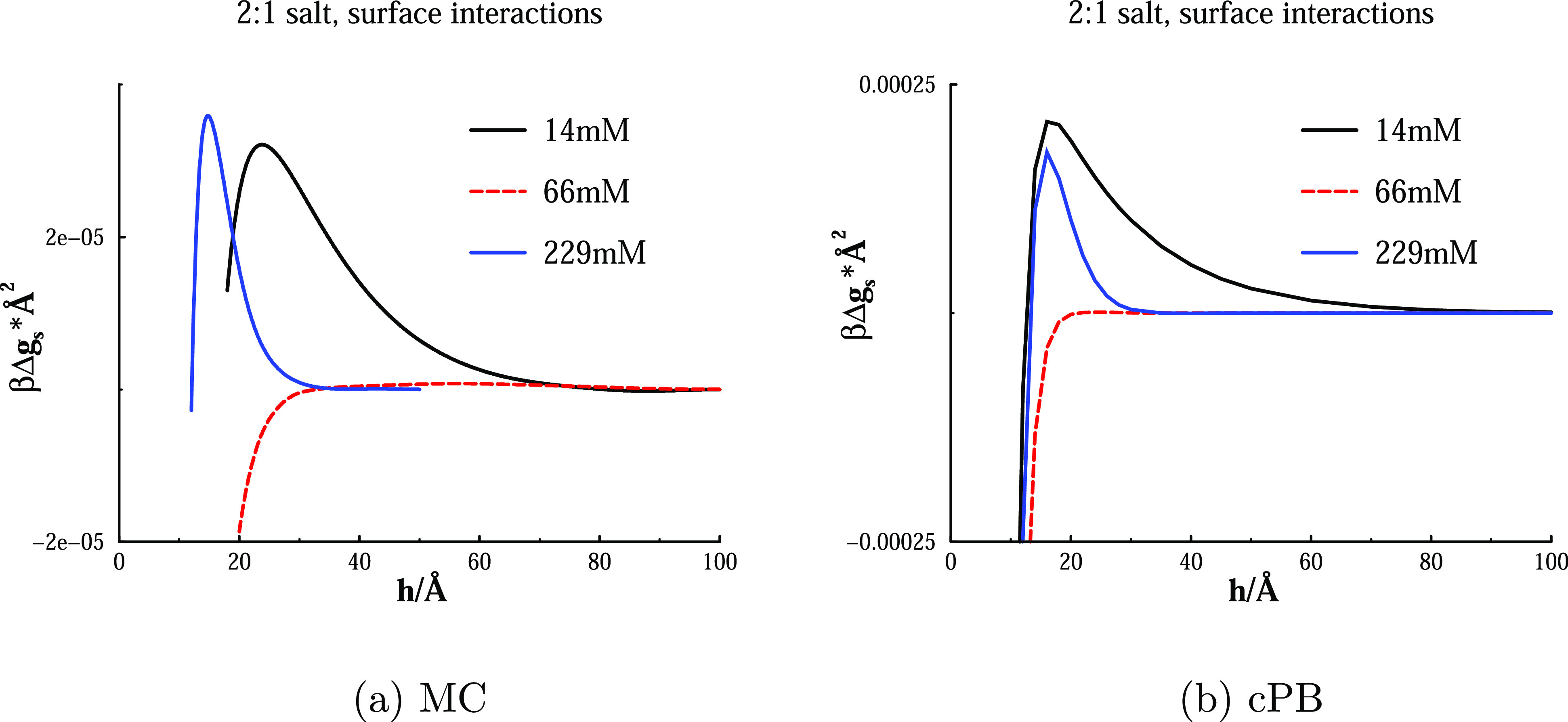
Variations of free energies per unit area with surface separation, as obtained from MC simulations and cPB calculations. Conditions as in Figure 7.

Finally, we turn our attention to the corresponding interaction free energies, displayed in Figure 8. We note the expected non-monotonic behaviour, where a long-ranged barrier at low concentrations is removed as the salt concentration is increased to the threshold value that corresponds to “perfect neutralization” (Figure 7). We have seen that a further increase generates overcharging and this manifests itself by the re-establishment of a free energy barrier. This barrier has a relatively short range, on account of an increased ionic screening, but can nevertheless reach high values. Experimentally, overcharging and the concomitant re-establishment of a repulsive barrier at high salt seems like a rare observation, that is, generally a more highly valent counterion is required. There are a number of possible reasons for this, such as: our use of rather small model ions, ion-specific adsorption (presumable of anions), a rare occurrence (in practice) of such highly charged surfaces, or the fact that we do not include any van der Waals attraction in our treatment.

It is gratifying to note that the cPB calculations are quite accurate also for this system, which deviates considerably from the 3:1 salt solution previously investigated. The overall tendency of cPB to overestimate how much the barrier height, as well as the apparent surface charge density, varies with concentration is found here too. This may possibly be related to the absence of any excluded volume interactions between the ions in the cPB treatment. However, the theory is extremely simple, and the improvement over standard PB is quite dramatic. The latter will not even qualitatively predict overcharging, or an attractive correlation regime, under *any* circumstances. It should be noted that transforming a PB code, using a DFT formulation, into its cPB correspondence only requires a few (less than 5) lines of code.

## Conclusions

This work demonstrates that experimental observations of overcharging, and its direct relation to the reformation of free energy barriers, can be captured by a very simple primitive model description, free of any bare surface heterogeneity or ion-specific adsorption. This does not mean that such aspects always are irrelevant, only that they are not required to explain the observed behaviours. We argue that the simple extension of PB to form cPB, whereby correlation effects are approximately managed, is useful and pedagogically appealing. Moreover, the mentioned experimental observations are qualitatively captured not only by simulations but also by the cPB calculations. We hope that this work can help to merge the seemingly disparate views of “ion correlation attraction” and “patch attraction” (or “heterogeneous surface attraction”).

## Supporting Information Available

Comparisons with the original formulation of the cPB (ZIP)
Simulation results obtained with 1:1 salt and comparisons with PB and cPB predictions (PDF)
