# Supplementary material for: Overcharging and Free Energy Barriers for Equally Charged Surfaces Immersed in Salt Solutions
Source: Langmuir. 2021 Dec 1;37(49):14360–8. doi: 10.1021/acs.langmuir.1c02268 (PMC8675215; doi:10.1021/acs.langmuir.1c02268)
Supplement: Supplementary file 1 — la1c02268_si_001.zip [file la1c02268_si_001.zip › Supporting_Information/SI.pdf]

Overcharging and free energy barriers, for equally charged surfaces immersed in salt solutions

Samuel Stenberg and Jan Forsman*

Theoretical Chemistry, P.O.Box 124, S-221 00 Lund, Sweden

E-mail: jan.forsman@teokem.lu.se,phone:int+46462220381

Supporting Information

Here, we report results using the original formulation of the cPB theory.[1] We also illustrate that standard PB performs reasonably well for 1:1 salts, in aqueous solutions.

Predictions by the original cPB

In the original cPB formulation,[1] R_c was approximated as:

$$R_c^{\alpha} = \sqrt{\frac{e}{\pi}\left|\frac{z_\alpha}{\sigma}\right|} \tag{S1}$$

This obviously leads to somewhat weaker correlation effects, compared to the version used in the main paper, where R_c is estimated as being 50% larger. A consequence of this is that the salt concentration needs to be higher, in order to generate overcharging. Nevertheless, the original cPB also predicts a monotonic attraction within a rather narrow salt concentration interval, where the surfaces are "perfectly neutralized". At higher and lower concentrations, significant free energy barriers develop, between surfaces that are effectively "undercharged", or "overcharged". This is summarized in Figure S1. We note that the concentration at which

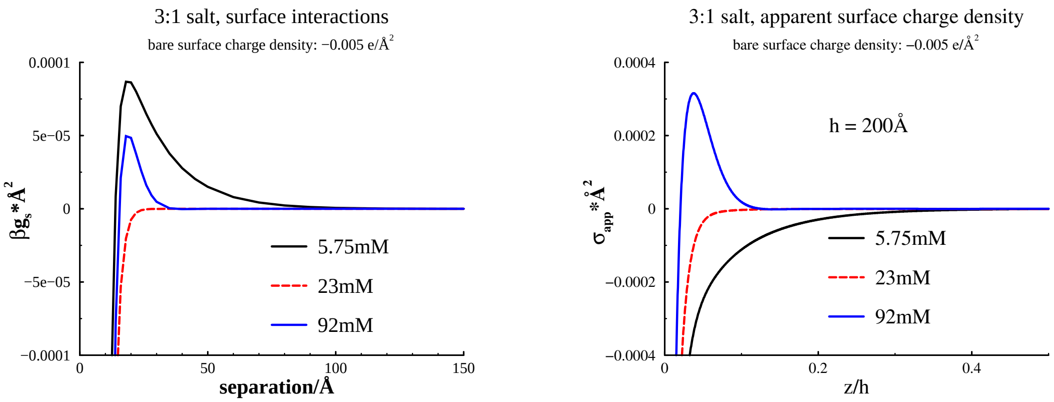

(a) Interaction free energies (b) Apparent surface charge density profiles

Figure S1: Predictions by the original cPB,[1] for 3:1 salts, in aqueous solutions ($\sigma_s = -0.005e/\text{Å}^2$).
(a) Interaction free energies.
(b) Apparent surface charge density profiles.

the surfaces are perfectly neutralized, and thus monotonically attract, is about 5 times higher than with the modified R_c estimate (main paper). But all qualitative aspects remain.

Interactions with 1:1 salts

Here, we have set $\sigma_s = -0.01e/\text{Å}^2$, i.e. the same surface charge density as with 2:1 salts, in the main paper. No charge reversal will occur for these conditions (1:1 salt), and the interactions are monotonically repulsive, and with a monotonic response (reduced range) to salt addition. In other words, a classic DLVO response, although we leave out van der waals attractions in our treatment. Under these circumstances, standard PB will perform reasonably well. We have chosen to illustrate this for a single bulk salt concentration (22.5 mM), and within a rather limited separation regime, in Figure S2 where we compare net pressure simulation data with cPB (main paper version), and PB predictions. The cPB still outperforms the PB, but the latter is nevertheless reasonably accurate, and qualitatively correct.

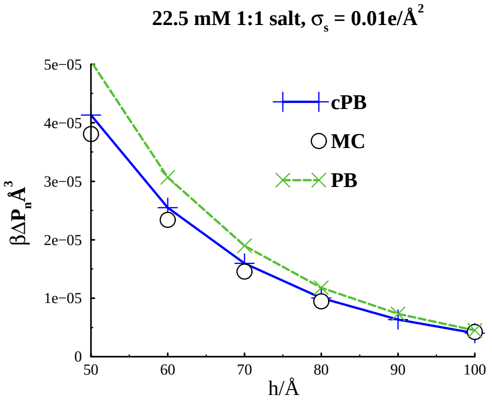

Figure S2: Net normal pressures, with 22.5mM 1:1 salt, as obtained by grand canonical simulations (MC), cPB calculations, and calculations with standard PB.

References

(1) Forsman, J. A simple correlation-corrected Poisson-Boltzmann theory. *The Journal of Physical Chemistry B* **2004**, *108*, 9236–9245.
