# Supplementary figures and images for: Overcharging and Free Energy Barriers for Equally Charged Surfaces Immersed in Salt Solutions
Source: Langmuir. 2021 Dec 1;37(49):14360–8. doi: 10.1021/acs.langmuir.1c02268 (PMC8675215; doi:10.1021/acs.langmuir.1c02268)

# 3:1 salt, surface interactions

bare surface charge density:  $-0.005 \text{ e}/\text{\AA}^2$

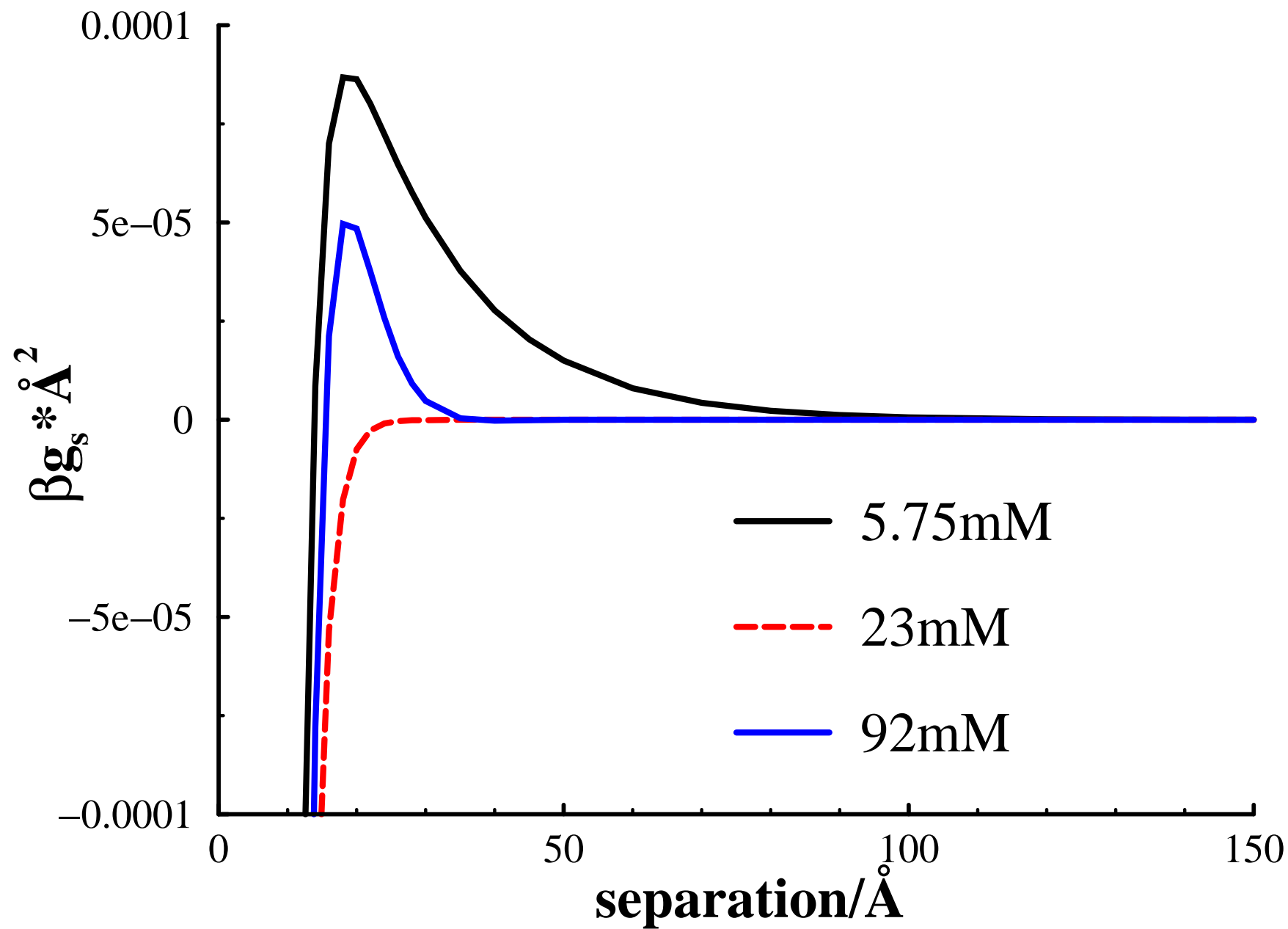

Supplement: Supplementary file 1 — la1c02268_si_001.zip [file la1c02268_si_001.zip › Supporting_Information/gs31_apm200_originalcPB.pdf]

# 3:1 salt, apparent surface charge density

bare surface charge density:  $-0.005 \text{ e}/\text{\AA}^2$

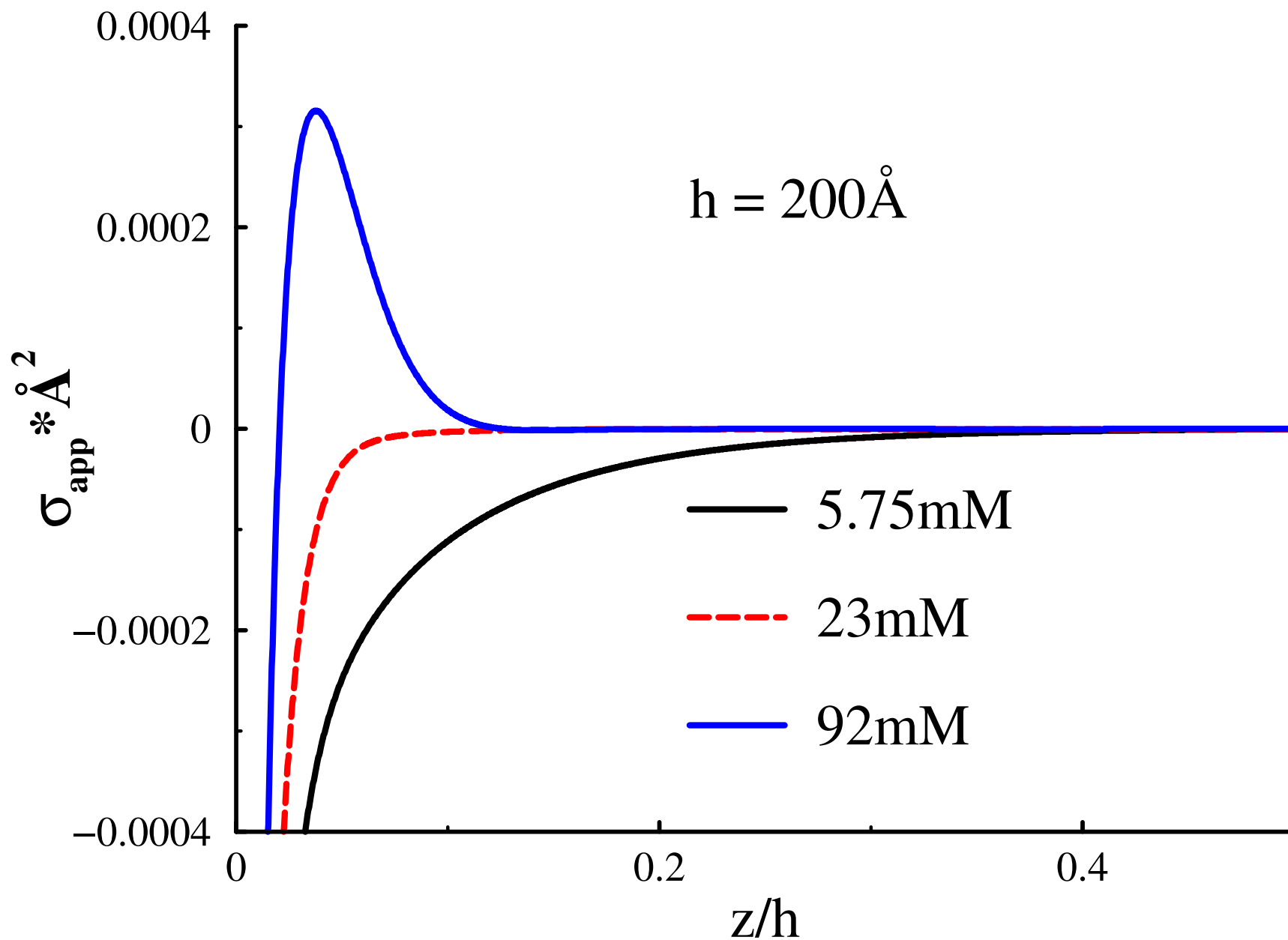

Supplement: Supplementary file 1 — la1c02268_si_001.zip [file la1c02268_si_001.zip › Supporting_Information/appsch31_apm200_originalcPB.pdf]

22.5 mM 1:1 salt,  $\sigma_s = 0.01\text{e}/\text{\AA}^2$

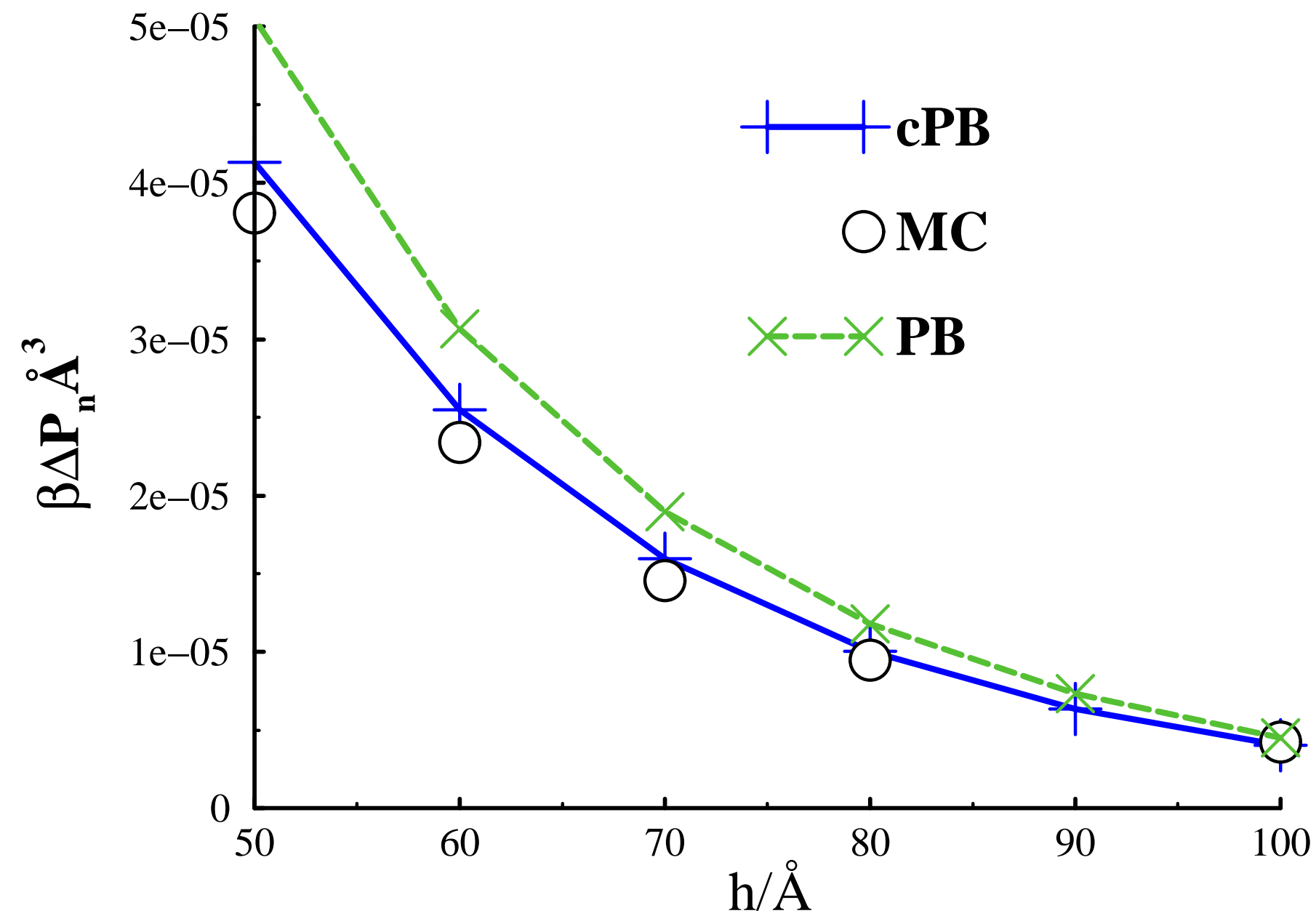

Supplement: Supplementary file 1 — la1c02268_si_001.zip [file la1c02268_si_001.zip › Supporting_Information/netpressure_monovalent.pdf]
